# Left atrial stiffness index – an early marker of left ventricular diastolic dysfunction in patients with coronary heart disease

**DOI:** 10.1186/s12872-024-04047-y

**Published:** 2024-07-17

**Authors:** Yingxuan Tu, Xin Liu, Xiaoqing Li, Na Xue

**Affiliations:** Department of Ultrasound, Baoding No.1 Central Hospital, Baoding, Hebei Province 071000 China

**Keywords:** Coronary heart disease, Left Atrial Stiffness Index, Left atrial automatic strain technique, Left ventricular diastolic function

## Abstract

**Aims:**

To evaluate the correlation between left atrial stiffness index (LASI) and left ventricular diastolic function in patients with coronary heart disease (CHD) by Autostrain LA technique.

**Methods:**

This was a retrospective analysis that included a total of 82 CHD patients who had suitable image quality for left atrial strain measurement. According to the 2016 ASE/EACVI guidelines for the echocardiographic assessment of diastolic dysfunction, the patients were divided into three groups: normal left ventricular diastolic function group (*n* = 26), indeterminate left ventricular diastolic function (*n* = 36), and left ventricular diastolic dysfunction (LVDD) (*n* = 20). The left atrial conduit strain (LAScd), Left atrial contractile strain (LASct), left atrial reservoir strain (LASr) and its derived parameters, including LASI and left atrial filling index (LAFI), were compared among the three groups. Furthermore, we conduct a correlation analysis between LASI and left ventricular diastolic function in patients with CHD.

**Results:**

LASr and LAScd in normal group were higher than those in indeterminate group, LASr and LAScd in indeterminate group were higher than those in LVDD group, LASI in normal group was lower than that in indeterminate group, and LASI in indeterminate group was lower than that in LVDD group (*P* < 0.001). LASct in both normal and indeterminate groups was higher than that in LVDD group (*P* < 0.05). The LAFI of normal group was lower than that of indeterminate group and LVDD group (*P* < 0.001). LASI was positively correlated with E/e’(*r* = 0.822) (*P* < 0.001). LASr and E/e’ were negatively correlated (*r* = -0.637) (*P* < 0.001).

**Conclusion:**

LASI is closely related to the changes of left ventricular diastolic function in CHD patients.

## Introduction

Coronary heart disease (CHD) is a heart disease caused by atherosclerosis of the coronary arteries, resulting in myocardial ischemia, hypoxia or necrosis from narrowing or occlusion of the coronary arteries. It has become one of the diseases seriously affecting people’s health worldwide. The final stage of CHD is heart failure, with the initial stage being left ventricular diastolic dysfunction (LVDD). LVDD is an important determinant of long-term survival and prognosis in CHD patients. Therefore, early assessment of left ventricular diastolic function and identification of LVDD is critical for clinical diagnosis and treatment [1, 2].

In 2016, ASE/EACVI updated the recommended guidelines for echocardiographic assessment of left ventricular diastolic function [3]. The guidelines proposed four recommended variables for identifying diastolic dysfunction and their abnormal cutoff values are annular e’ velocity (septal e’ < 7 cm/sec, lateral e’ < 10 cm/sec), average E/e’ ratio > 14, LA maximum volume index > 34 mL/m^2^, and peak TR velocity > 2.8 m/sec. LV diastolic function is normal if more than half of the available variables do not meet the cutoff values for identifying abnormal function. LV diastolic dysfunction is present if more than half of the available parameters meet these cutoff values. The study is inconclusive if half of the parameters do not meet the cutoff values. The atrium regulates ventricular filling function and maintains normal physiological perfusion of the ventricle through its storage function, conduit function, and pump function. Changes in left atrial (LA) function are important influencing factors for the occurrence of LVDD. Recently, many observational studies have confirmed that left atrial systolic strain is a new indicator for assessing left ventricular diastolic function [4].

Speckle tracking echocardiography has been widely used in clinical practice. However, due to the complexity of the left atrial geometry, traditional left ventricular measurement software cannot effectively evaluate left atrial function [5]. Real-time three-dimensional echocardiography and left atrial automatic strain technology (Autostrain LA) can accurately and quantitatively evaluate LA function [6–8]. This article aims to evaluate the corresponding strain and its derived parameters of left atrium in patients with CHD through Autostrain LA technology and explore their correlation with left ventricular diastolic function.

## Methods

### Patient population

This retrospective study selected 96 CHD patients who underwent routine echocardiography and coronary angiography at Baoding No.1 Central Hospital from January 2019 to December 2021. The patients were in sinus rhythm with normal resting wall motion and preserved left ventricular ejection fraction (LVEF) ≥ 50% having cardiac symptoms such as angina, ischemic-type chest pain, or other symptoms suggestive of myocardial ischemia. CHD was defined as > 50% luminal stenosis in one or more major epicardial vessels by coronary angiography examination. Exclusion criteria included LVEF < 50%, old myocardial infarction, left ventricular hypertrophy, valvular heart disease, pulmonary hypertension, Arrhythmia (including a history of atrial fibrillation, atrial flutter, or ventricular arrhythmia, etc.) and poor image quality. This study was approved by the hospital’s ethical committee [2022]027.

### Conventional transthoracic echocardiography

In our study, the Philips EPIQ 7C system, armed with the cardiac probe X5-1 (operating at 1.0–5.0 MHz), was employed for ultrasound imaging. Patients were positioned in the left lateral decubitus, with electrocardiogram monitoring in place. Within the parasternal long-axis view, key measurements were taken, including the left atrial diameter (LAD), left ventricular end-diastolic diameter (LVEDd), ventricular septal end-diastolic diameter (IVSDd), and left ventricular posterior wall end-diastolic diameter (LVPWDd). The left ventricular ejection fraction (LVEF) was subsequently derived through M-mode ultrasonography. Switching to the apical four-chamber view, we ascertained the tricuspid regurgitation velocity (TRV) and the peak early diastolic mitral inflow velocity (E). Furthermore, the mitral annular septal e’ and lateral wall e’ were evaluated using tissue Doppler imaging, allowing for the calculation of the average E/e’ ratio.

### Real-time three-dimensional echocardiography and left atrial automatic strain technology

**Fig. 1:**
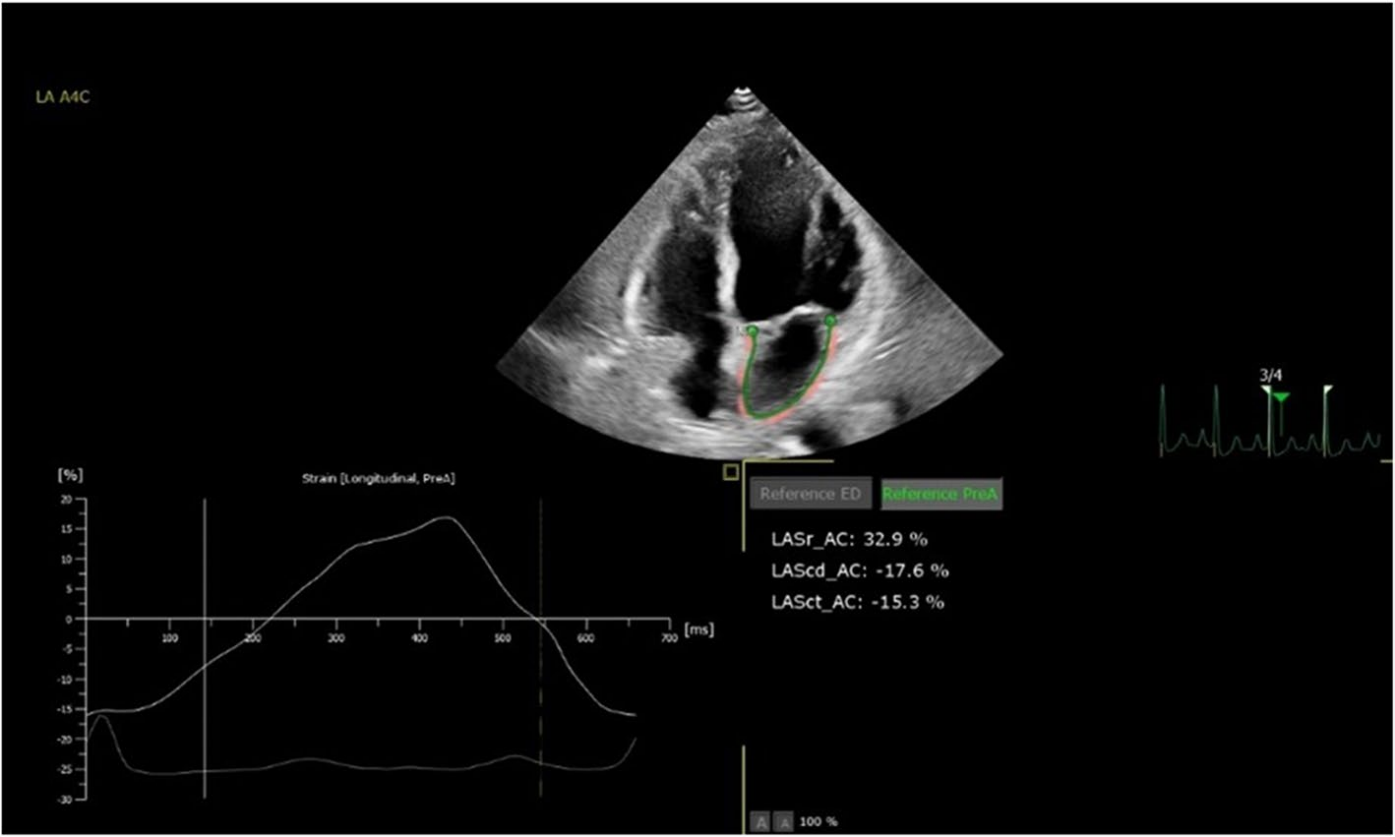
AutoStrain LA image from four-chamber apical view. Setting the starting point (Reference PerA) of strain analysis at the beginning of the p wave on the ECG allowed us to define first negative peak, first positive peak and the difference of these peaks which corresponded to atrial contractile strain (LASct), conduit strain (LAScd) and reservoir strain (LASr)

**Fig. 2:**
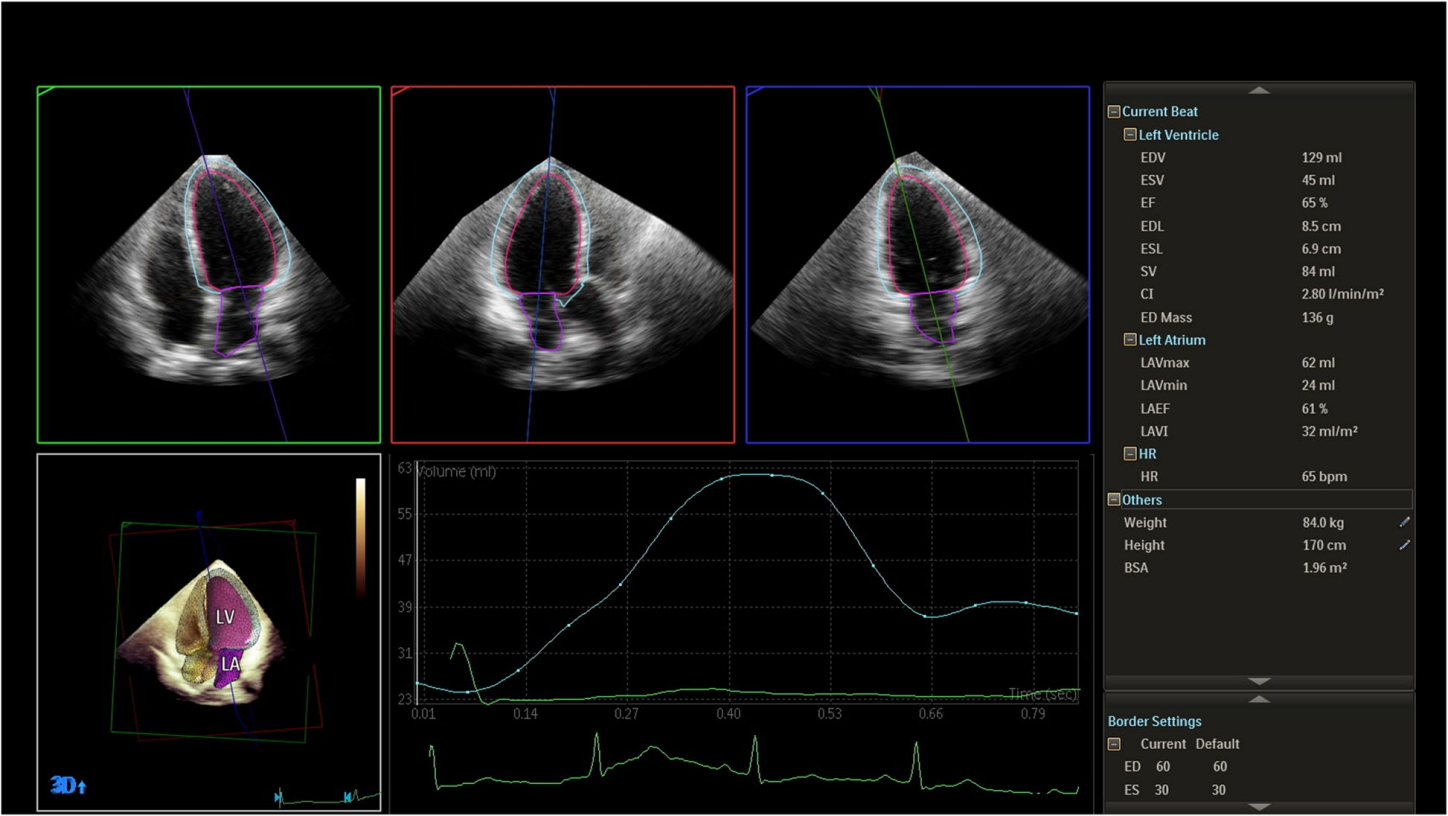
The LAVI calculated using the DHM function

Standard apical 4-chamber cardiac images were taken from the patients, and the patients were instructed to hold their breath at the end of inspiration. The 2D dynamic images were retained for four consecutive cardiac cycles or more, with a frame rate of > 40 frames/second. The two-dimensional dynamic images were analyzed by AutoStrain LA, which automatically traced the endocardial border and manually adjusted it if there was any deviation in the image tracing, and the average peak strain in each time phase of LA was measured and plotted on the strain curve. Using the ECG P-wave onset as the zero point, the first negative peak represented the LA contractile function (LASct), the first positive peak represented the LA conduit function (LAScd), and the difference between the two peaks represented the LA reservoir function (LASr) (Fig. 1). The Left Atrial Stiffness Index (LASI), LASI = (E/e’)/LASr and the Left Atrial Filling Index (LAFI), LAFI = E/LASr were calculated. Click on HM ACQ to enter the HM acquisition mode. Instruct the patient to hold their breath at the end of inhalation and capture a three-dimensional dynamic full-volume image that spans at least four beats, with a frame rate exceeding 40 frames per second. Proceed to the DHM function to analyze the captured three-dimensional dynamic full-volume image. Input the patient’s height and weight, and the software will automatically calculate the Left Atrial Volume Index (LAVI) (Fig. 2).

### Statistical Analysis

GraphPad Prism 8.0 and SPSS Statistics 25.0 software were used for statistical analysis. A Kolmogorov–Smirnov test was used to verify the normal distribution of variables. Continuous variables were reported as mean ± standard (SD) for normally distributed variables, while nonnormally distributed variables were reported as the median and interquartile range. The parameters that met the normality test were compared between groups by ANOVA and pair-to-group comparison by LSD test. If the variances were not homogeneous, Tamhani was selected for pair-to-group comparison. Kruskal–Wallis H test was used to compare parameters that did not conform to normal distribution. Statistical significance was tested using a χ^2^ test for categorical variables, Chi-square tests were used to compare groups. Spearman correlation coefficient method was used for correlation analysis. *P* value of < 0.05 was considered significant.

## Results

### Baseline characteristics

Ninety-six patients with stable CHD and preserved LVEF were included in this study. Wherein, 14 patients were excluded, including four patients with left ventricular hypertrophy, four patients with valvular heart disease, three patients with a history of atrial fibrillation, and three patients owing to poor image quality. Ultimately, 82 patients were included in the study. The patients ranged in age from 32 to 86 years old, with an average age of 51.7 ± 9.7 years. There were 42 males and 40 females. Based on the 2016 ASE/EACVI recommendations on echocardiographic assessment of cardiac diastolic function [3], 82 patients were grouped into three categories: 26 cases in the normal group, 36 cases in the indeterminate group, and 20 cases in the LVDD group. There were no statistically significant differences in general clinical data such as age, gender, height, weight, blood pressure, blood glucose, medical history, coronary angiography and heart rate among patients in each group (Table 1).

**Table 1:** clinical characteristics

Variable	normal group (*n* = 26)	indeterminate group (*n* = 36)	LVDD group (*n* = 20)	F/${\upchi }^{2}$	*P*
Age (years)	49.85 ± 7.29	52.56 ± 11.93	52.60 ± 7.75	0.699	0.5
male (%)	13(50)	20 (56)	9(45)	0.596	0.742
height (cm)	167.54 ± 6.79	167.97 ± 6.50	167.00 ± 6.77	0.139	0.871
Weight (kg)	70.31 ± 9.12	70.48 ± 10.10	68.39 ± 9.68	0.330	0.72
Systolic blood pressure(mmHg)	148.08 ± 14.95	144.03 ± 11.12	151.60 ± 13.52	2.270	0.11
Diastolic blood pressure(mmHg)	84.38 ± 7.23	89.92 ± 6.73	85.38 ± 6.37	0.890	0.415
blood glucose (mmol/L)	6.31 ± 0.97	6.84 ± 0.83	6.08 ± 0.92	0.798	0.454
Heart rate (beats/min)	76.31 ± 8.16	80.51 ± 8.92	81.00 ± 11.71	1.926	0.153
Medical history, *n* (%)					
HTN(%)	19(73.1)	23(63.8)	18(90.0)	4.465	0.107
DM(%)	8(30.7)	15(41.6)	10(50.0)	1.792	0.408
HLP(%)	14(53.8)	16(44.4)	14(70.0)	3.377	0.185
coronary angiography: vessel involved, *n* (%)					
Single vessel	14(53.8)	20(55.6)	8(40.0)	1.350	0.509
Multiple vessels	12(46.2)	16(44.4)	12(60.0)	1.350	0.509
Culprit vessel, *n* (%)					
LMCA	6(13.6)	9(13.6)	6(14.0)	3.272	0.774
LAD	13(29.5)	17(25.8)	14(32.6)	3.272	0.774
LCx	10(22.7)	24(36.4)	13(30.2)	3.272	0.774
RCA	15(34.1)	16(24.2)	10(23.3)	3.272	0.774

Data are expressed as mean ± SD or as number (percentage). *HTN* Hypertension, *DM* Diabetes Mellitus, *HLP* hyperlipidaemia, *LMCA* Left main coronary artery, *LAD* Left anterior descending artery, *LCx* Left circumflex artery, *RCA* Right coronary artery

#### General ultrasonic data

There were no statistically significant differences in LVEF, LVEDd, LVEDV, IVSDd, LVPWDd, and TR among all groups (*P* > 0.05). Additionally, no significant differences were observed in LAD, LAVI, mitral annular septum e’, mitral annular sidewall e’, and E/e’ between the normal and indeterminate groups (*P* > 0.05). However, the LVDD group exhibited significantly higher levels of LAD, LAVI, and E/e’ compared to the normal and indeterminate groups, with statistical significance (all *P* < 0.05). Conversely, the mitral annulus interval e’ and mitral annulus lateral wall e’ were significantly lower in the LVDD group compared to the other two groups (all *P* < 0.05) (Table 2).

**Table 2:** Echocardiographic variable comparison between groups

Variable	normal group (*n* = 26)	indeterminate group (*n* = 36)	LVDD group (*n* = 20)	F	P
LVEF	68.50 ± 4.51	67.53 ± 4.83	68.00 ± 4.65	0.327	0.722
LVEDd	44.54 ± 3.56	46.33 ± 3.26	44.50 ± 3.68	2.770	0.069
LVEDV	91.65 ± 16.57	99.25 ± 15.23	91.60 ± 17.40	2.233	0.114
IVSDd (mm)	9.15 ± 0.83	9.36 ± 0.90	9.20 ± 0.89	0.475	0.624
LVPWDd (mm)	9.23 ± 0.99	8.97 ± 1.16	9.20 ± 1.01	0.533	0.589
TR (cm/s)	231.15 ± 37.38	235.58 ± 38.45	236.40 ± 34.57	0.146	0.864
septum e’	8.37 ± 0.85	7.81 ± 1.29	4.85 ± 0.87^ab^	69.206	< 0.001
sidewall e’	10.92 ± 0.55	10.49 ± 1.57	8.86 ± 0.83^ab^	19.320	< 0.001
E/e’	11.13 ± 0.65	11.56 ± 1.16	14.57 ± 0.34^ab^	102.915	< 0.001
LAD(mm)	32.42 ± 3.24	33.44 ± 3.19	39.35 ± 1.27^ab^	37.836	< 0.001
LAVI(ml/m^2^)	30.62 ± 1.98	32.14 ± 4.52	35.85 ± 0.93^ab^	15.304	< 0.001

Data are expressed as mean ± SD. *LVEF* left ventricular ejection fraction, *LVEDd* left ventricular end-diastolic diameter, *LVEDV* left ventricular end-diastolic volume, *IVSDd* interventricular septal end-diastolic thickness, *LVPWDd* left ventricular posterior wall end-diastolic thickness, *TR* tricuspid regurgitation, *LAD* left atrial diameter, *LAVI* left atrial volume index

Compared with normal group, ^a^*p* < 0.05

Compared with indeterminate group, ^b^*p* < 0.05

#### Left atrial strain and its derived parameters

**Fig. 3:**
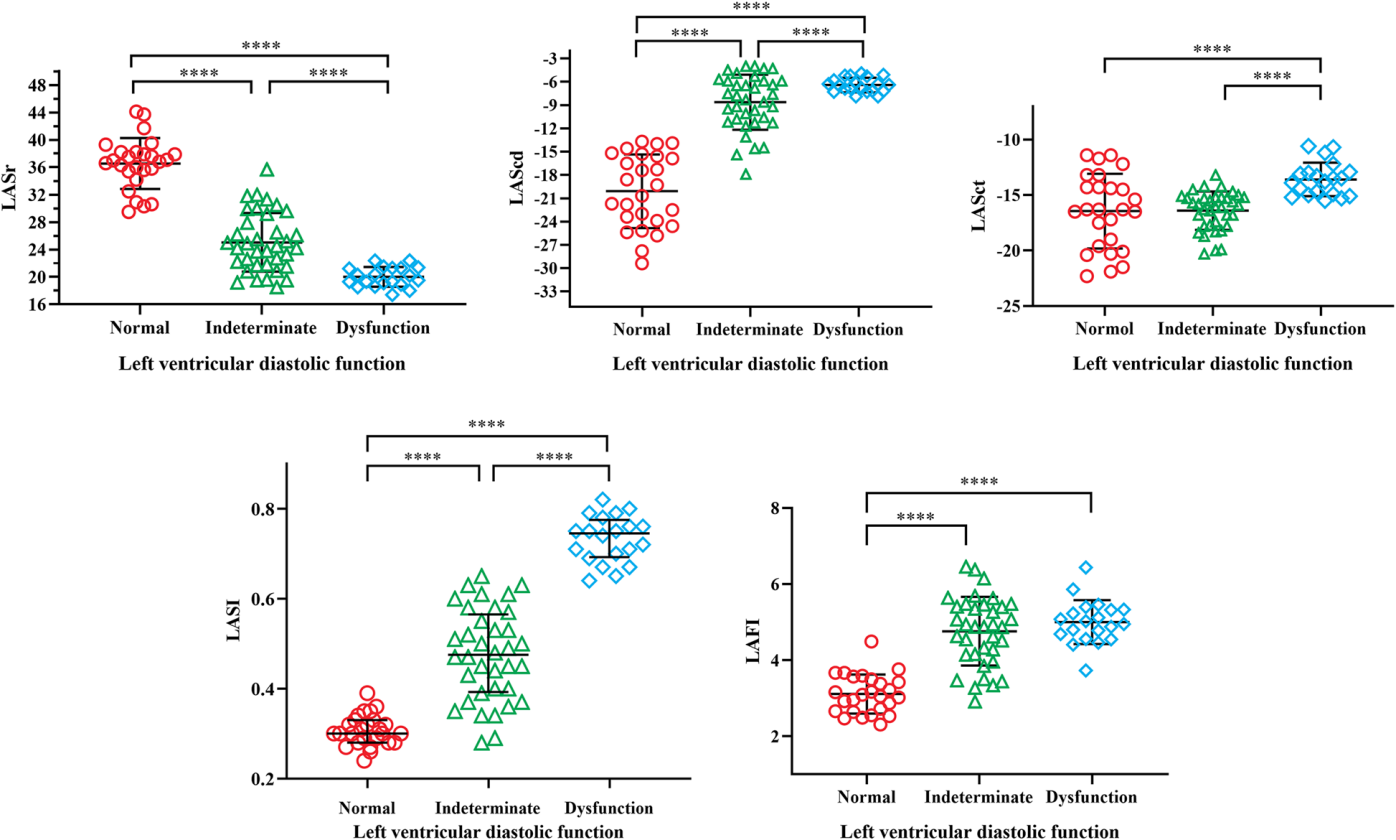
The comparison of differences in LASr, LAScd, LASct, LASI and LAFI among the groups

Compared among the three groups, LASr, LAScd, and LASI showed trend changes, and the differences were significant (*P* < 0.001): Specifically, the normal group exhibited higher LASr and LAScd values compared to the indeterminate group, and similarly, the indeterminate group showed higher LASr and LAScd values than the LVDD group. Conversely, the normal group had a lower LASI compared to the indeterminate group, and the indeterminate group had a lower LASI than the LVDD group (*P* < 0.001). There was no significant difference in LASct between normal group and indeterminate group (*P* > 0.05), and the LASct in normal group and indeterminate group was higher than that in LVDD group (*P* < 0.05). The LAFI of normal group was lower than that of indeterminate group and LVDD group, and the difference was statistically significant (*P* < 0.001), while the LAFI difference between indeterminate group and LVDD group was not statistically significant (*P* > 0.05) (Table 3). The comparison of differences in LASr, LAScd, LASct, LASI and LAFI among the groups was shown by Fig. 3.

**Table 3:** Left atrial strain and its derived parameters

Variable	normal group (*n* = 26)	indeterminate group (*n* = 36)	LVDD group (*n* = 20)	F/H	*P*
LASr (%)	36.55 ± 3.71	25.04 ± 4.28^a^	20.02 ± 1.44^ab^	133.665	< 0.001
LAScd (%)	-20.10 ± 4.74	-8.63 ± 3.54^a^	-6.43 ± 0.93^ab^	106.006	< 0.001
LASct (%)	-16.45 ± 3.36	-16.41 ± 1.73	-13.59 ± 1.51^ab^	11.170	< 0.001
LAFI	3.11 ± 0.51	4.76 ± 0.90^a^	5.00 ± 0.58^a^	51.899	< 0.001
LASI	0.30(0.28 ± 0.33)	0.48(0.39 ± 0.57)^a^	0.75(0.69 ± 0.78)^ab^	64.683	< 0.001

Data are expressed as mean ± SD or as median (25%–75% quartile). *LASr* left atrial reservoir strain, *LAScd* left atrial conduit strain, *LASct* left atrial contractile strain, *LAFI* left atrial filling index, *LASI* left atrial stiffness index

Compared with normal group, ^a^*p* < 0.05

Compared with indeterminate group, ^b^*p* < 0.05

### Correlation analysis

**Fig. 4:**
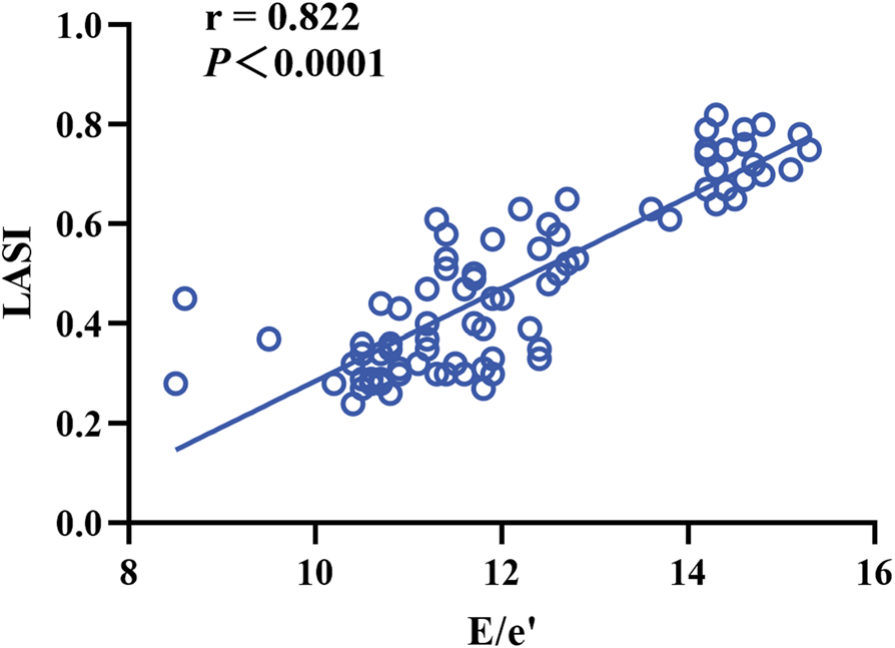
The correlation between LASI and E/e’

**Fig. 5:**
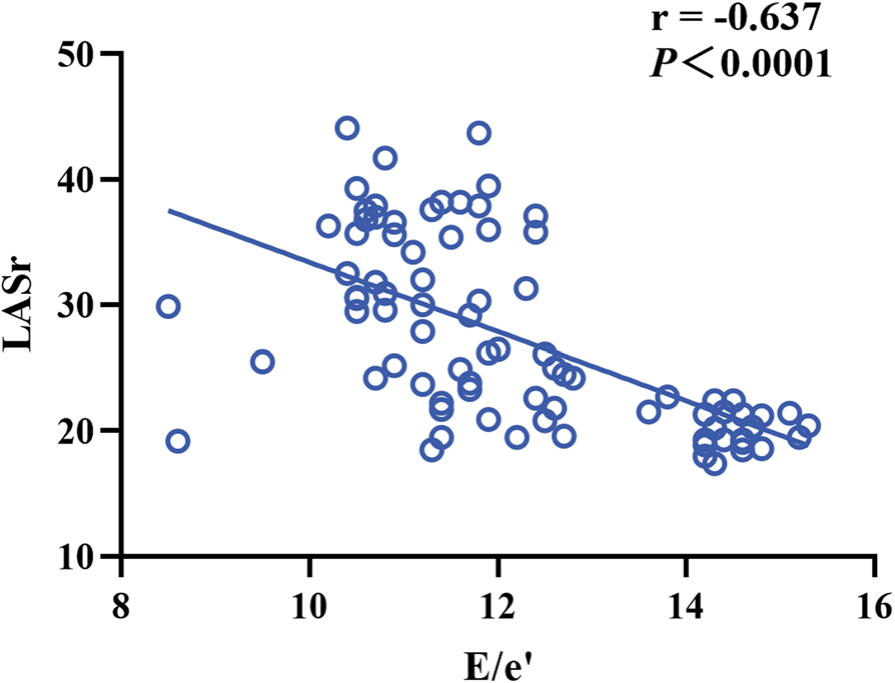
The correlation between LASr and E/e’

The correlation coefficients of LASI, LAFI, LASr, LAScd, LASct and E/e’, mitral septum e’, mitral lateral wall e’, E, LAVI are shown in Table 4. Among them, LASI is highly positively correlated with E/e’, *r* = 0.822. (Fig. 4) LASr showed a strong negative correlation with E/e’, *r* = -0.637, the difference was statistically significant (*P* < 0.001). (Fig. 5).

**Table 4:** Relationship between left atrial strain and left ventricular diastolic function

Variable	E/e’	septal e’	lateral e’	E	LAVI
LASI	0.822^b†^	-0.604^b†^	-0.519^b†^	-0.220^a&^	0.510^b†^
LAFI	0.507^b†^	-0.345^b&^	-0.177^#^	0.236^a&^	0.322^b&^
LASr	-0.637^b†^	0.560^b†^	0.406^b†^	0.176^#^	-0.469^b†^
LAScd	-0.539^b†^	0.456^b†^	0.285^b&^	0.065^#^	-0.387^b†^
LASct	0.430^b†^	-0.336^b&^	-0.288^b&^	-0.233^a&^	0.214^#^

^a^is correlated

^b^is significantly correlated

^&^*P* < 0.05

^†^*P* < 0.001

^#^*P* > 0.05

## Discussion

The final stage of CHD usually progresses to heart failure, and the initial stage of the disease development is often characterized by left ventricular diastolic dysfunction. Reduced left ventricular diastolic function is more sensitive to myocardial ischemia and appears earlier than reduced left ventricular systolic function. Therefore, early identification of LVDD in CHD patients is of great significance to improve clinical prognosis and reduce mortality.

In this study, grouping was conducted according to the guidelines [3]. The results indicated that when LVDD was definitively diagnosed based on the guidelines, the left ventricular diastolic function had already decreased, accompanied by structural changes in the left atrium. However, previous studies [9–12] have confirmed that functional changes in the left atrium precede structural changes. Functional impairment is a better indicator of the degree of pathological changes and remodeling in the left atrium compared to volumetric changes [13]. Therefore, in patients with uncertain diastolic function, there may be no significant changes in conventional structural parameters, while the left atrial function has already undergone alterations.

In addition, by comparing the left atrial function of the three groups of patients, it was found that for the part of patients with “gray area” in the guidelines, the differences of LASr, LAScd and LASI were statistically significant compared with the normal diastolic function group and the LVDD group and showed a trend change with the increase of the number of LVDD indicators. The left atrial function in the regulation of left ventricular filling pressure is mainly manifested in the following three phase functions: (1) reservoir function: receiving the blood returned by pulmonary veins during the systolic period; (2) conduit function: Blood flows into left ventricular via left atrial in the early stage of diastole; (3) contractile function: left atrial contraction at the end of diastole further promotes left ventricular filling [14]. The changes of left atrial reservoir function and conduit function occurred earlier in CHD patients, while the contractile function did not decrease significantly or even increased. This could be attributed to the fact that LASr and LAScd are primarily influenced by the longitudinal contractile of the LV and the compliance of the LA myocardium. As myocardial compliance is impaired, the filling pressure of the LV increases, leading to a rise in LA pressure. This, in turn, obstructs pulmonary venous return, resulting in a decrease in LASr and LAScd. However, LASct is mainly affected by the contractile function of the LA myocardium. In the early stages of LV diastolic dysfunction, compensatory changes may occur in the LA contractile function. Therefore, during the early stages of diastolic dysfunction, LASr and LAScd undergo changes while LASct remains relatively unchanged [15, 16]. Jingru Lin's research [17] discovered that LASr offered additional diagnostic value in the noninvasive assessment of LV filling pressures, aligning with certain aspects of our conclusions. Furthermore, through adept grouping based on established guidelines, we pioneered in exploring the correlation between LASI and left ventricular diastolic function. LASI and LAFI, as derived parameters of left atrial strain, reflect left atrial perfusion pressure and left ventricular function [18]. LASI, as a new index reflecting myocardial compliance, comprehensively considers the LA perfusion pressure and myocardial deformation ability and is not affected by passive traction of adjacent myocardial tissue and the amplitude of heart motion. It is more reliable and sensitive than traditional indicators [19]. It can be used to evaluate the compliance of the LA, reflect the stiffness of the LA, and has a good correlation with LV diastolic function. LAFI can further improve the efficiency of LASr in assessing the increase of left ventricular filling pressure. Therefore, when a patient has exactly two diastolic function indicators reaching the critical value, we can further evaluate the left atrial function and measure LASr, LAScd, and LASI to assess its left ventricular diastolic function more accurately.

Previous studies on left atrial strain mostly set the highest point of R wave of electrocardiogram as zero reference point [20–22].However, in order to better conform to the “physiology” of LA, this study used atrial cycle to evaluate LA function, that is, the zero reference point was set at the beginning point of p wave. The first negative peak of the strain curve appears at the end of LV diastolic period, representing the contractile function of LA (LASct). The first positive peak appears at the early stage of LV diastole, representing LA conduit function (LAScd), and the sum of absolute values of the two peaks represents LA reservoir function (LASr).

Different from ventricular muscle, atrial muscle is thinner and consists of two layers of muscle structure: the superficial layer and the deep layer. The superficial layer runs horizontally and surrounds the left and right atria, while the deep layer is divided into circular muscle and longitudinal muscle. The complex arrangement of left atrial muscle fibers determines its complex motion patterns [23]. Autostrain LA technology is an extension of two-dimensional speckle tracking imaging technology. Unlike previous studies that used left ventricular speckle tracking software to measure and analyze left atrial strain, Autostrain LA achieves time-resolved quantitative dynamic assessment of left atrial myocardial deformation by automatically tracking the motion trajectory of echo speckles that move synchronously with the left atrial myocardium, without being affected by sound beam angle or cardiac preload and afterload [24, 25]. Some studies have pointed out that the specialized left atrial strain analysis software significantly improves the intra-observer and inter-observer reproducibility [26].

There are some limitations in this study: LVDD was not graded to compare the correlation between each index and diastolic function in different grades of LVDD; Only LASr, LAScd, and LASI have been shown to evaluate LV diastolic dysfunction, but their diagnostic efficacy will be further improved in future studies; The lack of further exploration into the correlation between coronary artery lesion sites and parameters like LASI, as well as the relatively small number of cases.

## Conclusions

LA function in CHD patients is closely related to LV diastolic function. LASr, LAScd, and LASI have a certain reference value for evaluating LV diastolic function in patients when the guidelines cannot make a determination, providing imaging evidence for early clinical intervention measures, improving prognosis, and reducing mortality rates.

## Data Availability

The datasets used and/or analysed during the current study are available from the corresponding author on reasonable request.
